# Previous Intestinal Resection Is Associated with Postoperative Complications in Crohn's Disease: A Cohort Study

**DOI:** 10.1155/2020/2194382

**Published:** 2020-09-15

**Authors:** Yantao Duan, Yifan Liu, Yousheng Li

**Affiliations:** Department of General Surgery, Shanghai Ninth People's Hospital, Shanghai Jiao Tong University School of Medicine, Shanghai, China

## Abstract

**Background:**

To assess the influence of a previous intestinal resection on postoperative complications for Crohn's disease (CD).

**Methods:**

Data on patients with CD undergoing surgery in our department from January 2016 through December 2019 were retrospectively reviewed. Information collected included demographic details, surgical data, and postoperative outcome. A cross-sectional study design was employed. Associations between postoperative complications and preoperative clinical indicators were further analyzed.

**Results:**

Of the 129 patients with CD studied, 62 patients (48.06%) underwent previous resection. These patients were more likely to be older (*P* = 0.031), have longer disease duration (*P* = 0.025), use less 5-aminosalicylic acid/sulfasalazine preoperatively (*P* = 0.013), have lower body mass index (*P* = 0.003), and have a higher American Society of Anesthesiologists (ASA) Physical Status Classification System score (*P* = 0.043). Patients who had previous surgery had a longer duration of operation (*P* = 0.003), greater estimated blood loss (*P* = 0.001), and longer hospital stay (*P* < 0.001) and were more inclined to develop postoperative complications (*P* = 0.047), particularly anastomotic leak (*P* = 0.021) and severe (Clavien–Dindo grade III/IV) complications (*P* = 0.038). After multivariate analysis, previous intestinal resection (*P* = 0.019), preoperative use of steroids (*P* = 0.026), and ASA score of more than II (*P* < 0.001) were determined to be the independent prognostic risk factors for postoperative complications. During the 30-day follow-up period, there was no postoperative mortality or readmission.

**Conclusions:**

Previous intestinal resection in patients with CD is an independent predictor of overall postoperative complications.

## 1. Introduction

Crohn's disease (CD) is a chronic bowel inflammatory disease, mainly affecting the digestive tract, that frequently shows clinical symptoms in a relapsing and remitting manner [1]. The incidence of CD is higher in developed countries; however, with the development of its economy, China now is witnessing an increasing incidence of CD [2]. The aim of the therapeutic strategies is to maintain remission, with the purpose of preventing complications and disease progression.

Despite medical progress in the therapy for CD, the surgical risk rate after 1, 5, and 10 years from the diagnosis was reported to be about 16%, 33%, and 45%, respectively [3]. According to reports on the natural history of the disease, more than half of the patients with CD will undergo more than one surgery in their lifetime [4]. Although surgery is an effective way to remove the intestinal lesions and relieve symptoms, surgical resection is not a curative method for CD. Postoperative complications are very common, including anastomotic leakage, fistula formation, bowel obstruction, intestinal bleeding, and surgical site infection (SSI) [5, 6]. The key medications applied in the current treatment of CD, including immunomodulators, biologic drugs, and steroid hormones, are related to an increased incidence of postoperative complications after CD surgery [7, 8].

Preoperative treatment is necessary for patients with CD, including drainage of abdominal abscesses or intestinal fistulas, provision of nutritional support, induction of remission of CD, and use of biological agents [9]. Retrospective studies have tried to evaluate the risk factors for postoperative complications and have reported that advanced age, lower body mass index (BMI), poor nutritional status, higher American Society of Anesthesiologists (ASA) Physical Status Classification System score (ASA score), and the CD complications of abscess or intestinal fistula formation were related to increased postoperative complications [10–14]. Patients' overall condition has been improved through preoperative treatment and perioperative surgical home practice according to our previous research [15]. Because of the high CD recurrence rate and their complications, more than 50% of patients with CD will experience surgical recurrence within 10 years after the first operation [16]. However, the specific impact of the previous intestinal resection on the postoperative complications in patients with CD was still uncertain. To evaluate the influence of previous intestinal resection on postoperative complications of CD, we reviewed patients with CD who underwent surgery in our center. We also assessed the risk factors for postoperative complications in these patients.

## 2. Materials and Methods

### 2.1. Study Population

A cross-sectional study design was employed. The data of patients with CD were gathered from a retrospectively established database of a tertiary center from January 2016 through December 2019. Included in the current study were a total of 129 consecutive patients with CD who underwent surgical treatment at our Department of General Surgery. Patients with previous surgery for perianal disease, drainage of an abdominal abscess, or planned secondary surgery were excluded from this study. The study was approved by the Institutional Review Board of the Shanghai Ninth People's Hospital, Shanghai Jiao Tong University School of Medicine (Reference number: 2018297).

### 2.2. Clinical Assessment

Details from a prospective patient database comprising clinical data, including population demographics, preoperative case features, surgery-related characteristics, and postoperative outcome, were analyzed retrospectively. A standardized form was applied to acquire patient profiles, including detailed information on their illness and any surgery-related complications. Sex, age, BMI, and disease duration after the initial diagnosis of CD were all retrieved. Records were also collected regarding the disease characteristics, including Montreal classification for inflammatory bowel diseases, previous CD-related intestinal resection, and perianal lesions. The following surgery-related data were collected: ASA score, indications for surgery, intraoperative findings, estimated intraoperative blood loss, and estimated operative time. The type of procedure (open vs. conversion vs. laparoscopic approach) was also recorded. Preoperative medication records included the use of steroids, 5-aminosalicylic acid/sulfasalazine, immunomodulators, and biologic agents.

### 2.3. Assessment of Postoperative Complications

The primary endpoint of the study was the overall postoperative complications. Postoperative complications were defined as deviations that occurred from the routine postoperative procedure using the Clavien–Dindo classification system [17]. Specific 30-day postoperative complications including SSI, anastomotic leak, urinary tract infection (UTI), and pneumonia were recorded. The occurrence of ileus was also noted; it was defined as abnormal postoperative bowel function recovery or the use of a nasogastric tube because of vomiting without evidence of mechanical bowel obstruction [18]. Postoperative small-bowel obstruction (SBO) was considered to be the existence of mechanical intestinal obstruction symptoms associated with supporting findings on imaging studies [19, 20]. In addition, other parameters including dehydration, acute renal failure (ARF), and reoperation were noted. Hospital readmission was reviewed if it occurred within 30 days after discharge from the hospital.

### 2.4. Statistical Analysis

Continuous data were shown as mean ± standard deviation (SD) and were analyzed by the independent Student *t*-test or Mann–Whitney *U* test. The Kolmogorov–Smirnov test was used to test the normality of the data [21]. The categorical variables were reported as absolute frequencies and were analyzed by Pearson's chi-squared test or Fisher's exact test. Statistically significant factors by univariate analysis were further tested to find the independent factors related to postoperative complications by a multivariate logistic regression model. Two-tailed *P* values < 0.05 were considered to be statistically different. Statistical analyses were conducted with IBM SPSS Statistics for Windows, version 22.0 (IBM Corp., Armonk, New York, USA).

## 3. Results

### 3.1. Patient Demographics

A total of 129 patients with CD who underwent abdominal operations were included in the study; of these, 62 (48.06%) patients had a previous bowel resection. Characteristics of the patients in the two groups are detailed in Table 1. The mean (SD) age of patients who had undergone previous resection by the time of surgery was 40.56 (9.65) years, older than the 37.12 (8.31) years of the patients who had not undergone previous resection (*P* = 0.031). The mean (SD) duration of CD from the time of diagnosis in the group that had undergone previous resection was 8.45 (4.08) years, longer than the 6.85 (3.89) years in the group having no previous resection (*P* = 0.025). The mean (SD) BMI of the previous resection group was 17.67 (1.07), lower than the 18.27 (1.14) in the group having no previous resection (*P* = 0.003). The main clinical manifestations of the disease were ileocolonic location and penetrating disease behavior, which were confirmed in about 45% of patients with CD. The patients who had undergone previous resection were more likely to use less 5-aminosalicylic acid/sulfasalazine preoperatively (*P* = 0.013). Patients with previous CD-related bowel resection were associated with an increased ASA score (*P* = 0.043). The two groups showed no statistically significant difference in terms of sex, disease behavior, disease location, perianal disease, or preoperative use of medications (biologics, steroids, or immunomodulators). Most of the patients underwent surgery mainly for bowel fistula that could have progressed from CD-related bowel disease such as abscess or stricture.

Table 2 shows the surgical variables of the two patient groups. The most common surgery conducted in the group with no previous intestinal resection was ileocolonic resection, followed by small-bowel resection and segmental colectomy. However, the most common course in the previous resection group was small-bowel resection followed by ileocolonic resection and segmental colectomy (*P* = 0.026). Compared with the patients in the previous resection group, more patients with CD in the group with no previous resection underwent laparoscopic surgery (*P* = 0.031). The mean (SD) duration of operation in the previous resection group was 130.76 (27.58) min, longer than that of 117.43 (21.86) min in the group with no previous resection (*P* = 0.003). Patients with previous resection had a significantly greater estimated intraoperative blood loss than those without previous resection (mean [SD], 159.18 [59.06] mL vs. 129.33 [42.68] mL; *P* = 0.001). There were no statistical differences with regard to intraoperative findings of bowel fistulas and abscesses between the two groups.

### 3.2. Relationship between Previous Resection and Postoperative Complications

The postoperative complications within 30 days compared between the two groups are shown in Table 3. There were no deaths or readmissions. The total postoperative complication rate of our cohort was 42.64%. Patients of the previous resection group were associated with an increased rate of overall postoperative morbidity, especially anastomotic leak (*P* = 0.021) and severe postoperative complications (Clavien–Dindo grade III/IV) (*P* = 0.038). Meanwhile, there was no statistical significance in terms of SSI rate, UTI, pneumonia, SBO, ileus, dehydration, or ARF compared with patients without previous resection. Patients receiving previous resection had a statistically longer hospital stay than those without previous resection (mean [SD], 13.21 [6.68] days vs. 9.49 [4.06] days; *P* < 0.001).

### 3.3. Predictors of Postoperative Complications

In the current study, previous intestinal resection, increased ASA score, increased estimated blood loss, preoperative use of steroids, and longer hospital stay were all demonstrated to be associated with postoperative morbidity by univariate analysis (Table 4). Furthermore, three independent prognostic risk factors for postoperative complications were previous intestinal resection (OR = 1.621; 95% CI = 1.017–2.453; *P* = 0.019), preoperative use of steroids (OR = 1.597; 95% CI = 1.086–2.347; *P* = 0.026), and ASA score of more than II (OR = 1.854; 95% CI = 1.271–2.705; *P* < 0.001) (Table 5).

## 4. Discussion

As far as we know, the current study is the first to evaluate the risk factors of complications after reoperation for patients with CD. Our results indicate that patients with CD who had undergone previous surgery were more inclined to be older and have the following: lower BMI, longer disease duration since diagnosis, less use of 5-aminosalicylic acid/sulfasalazine, and higher ASA score. The previous intestinal resection increased the risk of postoperative morbidity within 30 days for patients with CD after surgery, especially anastomotic leak and severe postoperative complications (Clavien–Dindo grade III/IV).

Despite great progress in the medical treatment for CD, accompanied by the sequential introduction of steroids, immunomodulators, and biologics, more than half of patients with CD eventually have to undergo surgical treatment over the course of the disease [22]. The repeat intestinal resection rate in our cohort was 48%, which is similar to other studies showing a 25–45% rate of repeat resection [23]. The short-term outcomes after surgery for CD have been markedly improved by better perioperative treatment. However, postoperative morbidity still occurs in up to 40% of patients after surgery [24]. The current study emphasizes that repeat intestinal resection is associated with a significant increase in postoperative complications, particularly anastomotic leak. Patients with CD undergoing initial resection had a 5% incidence of anastomotic leak, similar to the rates published previously [25, 26]. However, repeat resection dramatically increased the leak rate to 19% compared with patients undergoing initial resection, leading to a threefold increase in anastomotic leak rates. The findings of this study support previous resection as a risk factor for anastomotic leak in patients with CD. Repeat resection may be an alternative marker for a more aggressive disease form, more complicated surgery, or altered vascular distribution. Repeat resection may suggest a prolonged dissection because of the atypical planes of bowel dissection, leading to increased risk of accidental enterotomy and additional bowel devascularization. Meanwhile, our study has also shown that operative time and the length of hospital stay of patients with CD undergoing primary intestinal resection were shorter than for those who underwent reoperation. Estimated blood loss appeared to be smaller in the group with no previous resection. The mortality and readmission rates were zero.

Studies have reported that preoperative antitumor necrosis factor therapy, preoperative steroids, preoperative poor nutritional status, recurrent CD, immunosuppressant medications, and the perforating CD phenotype were identified as risk factors for postoperative complications in CD [12, 27, 28]. In this study, we tried to determine the risk factors associated with postoperative morbidity. For overall complications, using univariate analysis in addition to previous intestinal resection, preoperative corticosteroids, higher ASA score, and greater estimated blood loss were also related to an increased rate of postoperative morbidity. Furthermore, previous intestinal resection, preoperative use of steroids, and ASA score higher than II were determined to be independent predictors of postoperative complications by multivariate analysis.

We acknowledge that this study has certain limitations. First, our current study was not designed as randomized control research and only included patients from a single center. All the patients who were enrolled were surgical patients who might have had greater disease severity, and the patients who had undergone previous intestinal resection might be more prevalent in our center. Therefore, there may be selection bias. Second, preoperative medication use was determined from medical records in our department. Although we did our best to carefully scrutinize all the available medication records, there may be bias. Finally, laboratory studies, including preoperative level of C-reactive protein, erythrocyte sedimentation rate, and albumin level, which might have a significant impact on postoperative complications, were also not analyzed in this study. Additional prospective clinical studies with more patients should be performed to further evaluate the results of our current study.

## 5. Conclusions

In conclusion, the current study has demonstrated that previous intestinal resection in patients with CD is a risk factor for postoperative complications. Furthermore, this study revealed that previous intestinal resection particularly increased the risk for anastomotic leak and severe postoperative complications. The findings should be incorporated into future surgical decision-making, especially with respect to the indications of intestinal resection and anastomosis.

## Figures and Tables

**Table 1:** Patient demographics and preoperative interventions.

Characteristics	No previous resection (*n* = 67)	Previous resection (*n* = 62)	*P* value
Male, no. (%)	38 (56.72)	28 (45.16)	0.190
Age at surgery, mean (SD) (y)	37.12 (8.31)	40.56 (9.65)	0.031
BMI, mean (SD)	18.27 (1.14)	17.67 (1.07)	0.003
Disease duration since diagnosis, mean (SD) (y)	6.85 (3.89)	8.45 (4.08)	0.025
Disease behavior, no. (%)			0.708
B1: nonstricturing, nonpenetrating	15 (22.39)	11 (17.74)	
B2: stricturing	24 (35.82)	21 (33.87)	
B3: penetrating	28 (41.79)	30 (48.39)	
Disease location, no. (%)			0.791
L1: terminal ileum	23 (34.33)	19 (30.65)	
L2: colon	15 (22.39)	13 (20.97)	
L3: ileocolon	25 (37.31)	27 (43.55)	
L4: upper gastrointestinal tract	4 (5.97)	3 (4.84)	
Perianal disease, no. (%)	14 (20.90)	17 (27.42)	0.386
Preoperative descriptions, no. (%)			
Biologics	15 (22.39)	21 (33.87)	0.146
5-ASA/sulfasalazine	25 (37.31)	11 (17.74)	0.013
Steroids	18 (26.87)	16 (25.81)	0.891
Immunomodulators	14 (20.90)	17 (27.42)	0.386
Indications for surgery, no. (%)			0.945
Unresponsiveness to medical management	13 (19.40)	11 (17.74)	
Bowel fistula(e) with or without disease-related abscess(es)	26 (38.81)	24 (38.71)	
Fibrosis/stricturing	23 (34.33)	23 (37.10)	
Other	5 (7.46)	4 (6.45)	
ASA score, no. (%)			0.043
1	17 (25.37)	9 (14.52)	
2	36 (53.73)	27 (43.55)	
3	12 (17.91)	22 (35.48)	
4	2 (2.99)	4 (6.45)	

BMI: body mass index; 5-ASA: 5-aminosalicylic acid; ASA: American Society of Anesthesiologists.

**Table 2:** Surgical data.

Characteristics	No previous resection (*n* = 67)	Previous resection (*n* = 62)	*P* value
Surgical procedures, no. (%)			0.026
Small-bowel resection	17 (25.37)	28 (45.16)	
Ileocolic resection	30 (44.78)	14 (22.58)	
Segmental colectomy	9 (13.43)	12 (19.35)	
Other (ostomy closure, strictureplasty, and stoma creation without resection)	11 (16.42)	8 (12.90)	
Surgical technique, no. (%)			0.031
Laparoscopy	38 (56.72)	22 (35.48)	
Open surgery	19 (28.36)	21 (33.87)	
Conversion	10 (14.93)	19 (30.65)	
Intraoperative findings, no. (%)			
Fistula(e)	22 (32.84)	25 (40.32)	0.377
Abscess(es)	17 (25.37)	14 (22.58)	0.711
Duration of operation, mean (SD) (min)	117.43 (21.86)	130.76 (27.58)	0.003
Estimated blood loss, mean (SD) (mL)	129.33 (42.68)	159.18 (59.06)	0.001

**Table 3:** Postoperative outcomes.

Outcome	No previous resection (*n* = 67)	Previous resection (*n* = 62)	*P* value
Overall complications, no. (%)	23 (34.33)	32 (51.61)	0.047
SSIs, no. (%)	13 (19.40)	16 (2.58)	0.384
Superficial incisional	7 (10.45)	8 (12.90)	0.664
Deep incisional	3 (4.48)	4 (6.45)	0.710
Organ or space infection	3 (4.48)	4 (6.45)	0.710
Anastomotic leak, no. (%)	4 (5.97)	12 (19.35)	0.021
UTI, no. (%)	1 (1.49)	2 (3.23)	0.608
Pneumonia, no. (%)	2 (2.99)	3 (4.84)	0.671
SBO, no. (%)	6 (8.96)	9 (14.52)	0.325
Ileus, no. (%)	10 (14.93)	12 (19.35)	0.504
Dehydration, no. (%)	2 (2.99)	3 (4.84)	0.671
Acute renal failure, no. (%)	1 (1.49)	3 (4.84)	0.350
Severe postoperative complications (Clavien–Dindo grade III/IV), no. (%)	7 (10.45)	15 (24.19)	0.038
Length of hospital stay, mean (SD) (d)	9.49 (4.06)	13.21 (6.68)	<0.001

SSI: surgical site infection; UTI: urinary tract infection; SBO: small-bowel obstruction.

**Table 4:** Risk factors associated with postoperative complications.

Variables	Unremarkable postoperative course (*n* = 74)	Any postoperative complications (*n* = 55)	*P* value
Univariate analysis			
Previous intestinal resection for CD, no. (%)	29 (39.19)	33 (60.00)	0.019
Male, no. (%)	36 (48.65)	30 (54.55)	0.508
Age at surgery, mean (SD) (y)	38.90 (9.96)	38.60 (7.90)	0.851
BMI, mean (SD)	18.10 (1.20)	17.82 (1.06)	0.171
Disease duration since diagnosis, mean (SD) (y)	7.37 (4.45)	7.94 (3.45)	0.433
Disease behavior, no. (%)			0.712
B1: nonstricturing, nonpenetrating	14 (18.92)	12 (21.82)	
B2: stricturing	28 (37.84)	17 (30.91)	
B3: penetrating	32 (43.24)	26 (47.27)	
Disease location, no. (%)			0.211
L1: terminal ileum	22 (29.73)	20 (36.36)	
L2: colon	13 (17.57)	15 (27.27)	
L3: ileocolon	34 (45.95)	18 (32.73)	
L4: upper gastrointestinal tract	5 (6.76)	2 (3.64)	
Perianal disease, no. (%)	16 (21.62)	15 (27.27)	0.458
Preoperative descriptions, no. (%)			
Biologics	17 (22.97)	19 (34.55)	0.147
5-ASA/sulfasalazine	16 (21.62)	20 (36.36)	0.065
Steroids	14 (18.92)	20 (36.36)	0.026
Immunomodulators	16 (21.62)	15 (27.27)	0.458
Indications for surgery, no. (%)			0.302
Unresponsiveness to medical management	10 (13.51)	14 (25.45)	
Bowel fistula(e) with or without disease-related abscess(es)	32 (43.24)	18 (32.73)	
Fibrosis/stricturing	26 (35.14)	20 (36.36)	
Other	6 (8.11)	3 (5.45)	
ASA score, no. (%)			0.003
1	20 (27.03)	6 (10.91)	
2	39 (52.70)	24 (43.64)	
3	12 (16.22)	22 (40.00)	
4	3 (4.05)	3 (5.45)	
Surgical procedures, no. (%)			0.259
Small-bowel resection	27 (36.49)	18 (32.73)	
Ileocolic resection	29 (39.19)	15 (27.27)	
Segmental colectomy	9 (12.16)	12 (21.82)	
Other (ostomy closure, strictureplasty, stoma creation without resection)	9 (12.16)	10 (18.18)	
Surgical technique, no. (%)			0.139
Laparoscopy	39 (52.70)	21 (38.18)	
Open surgery	18 (24.32)	22 (40.00)	
Conversion	17 (22.97)	12 (21.82)	
Intraoperative findings, no. (%)			
Fistula(e)	23 (31.08)	24 (43.64)	0.143
Abscess(es)	15 (20.27)	16 (29.09)	0.246
Duration of operation, mean (SD) (min)	121.72 (26.04)	126.69 (24.85)	0.276
Estimated blood loss, mean (SD) (mL)	133.85 (43.49)	156.89 (61.90)	0.014
Length of hospital stay, mean (SD) (d)	9.34 (2.87)	13.88 (7.46)	<0.001

CD: Crohn's disease; BMI: body mass index; 5-ASA: 5-aminosalicylic acid: ASA: American Society of Anesthesiologists.

**Table 5:** Multivariate analysis of risk factors for postoperative complications.

Variables	Odds ratio (95% CI)	*P* value
Estimated blood loss	1.407 (1.005-1.969)	0.081
Steroids	1.597 (1.086-2.347)	0.026
Previous intestinal resection for CD	1.621 (1.071-2.453)	0.019
ASA score	1.854 (1.271-2.705)	<0.001

CD: Crohn's disease; CI: confidence interval; ASA: American Society of Anesthesiologists.

## Data Availability

The datasets used in the current study are available from the corresponding author on reasonable request.
